# Structural properties of Al-rich AlInN grown on *c*-plane GaN substrate by metal-organic chemical vapor deposition

**DOI:** 10.1186/1556-276X-9-628

**Published:** 2014-11-23

**Authors:** Pei-Yin Lin, Jr-Yu Chen, Yi-Sen Shih, Li Chang

**Affiliations:** 1 Department of Materials Science and Engineering, National Chiao Tung University, Hsinchu 30010, Taiwan

**Keywords:** Metal-organic chemical vapor deposition, Epitaxy, AlInN, GaN

## Abstract

The attractive prospect for AlInN/GaN-based devices for high electron mobility transistors with advanced structure relies on high-quality AlInN epilayer. In this work, we demonstrate the growth of high-quality Al-rich AlInN films deposited on *c*-plane GaN substrate by metal-organic chemical vapor deposition. X-ray diffraction, scanning electron microscopy, and scanning transmission electron microscopy show that the films lattice-matched with GaN can have a very smooth surface with good crystallinity and uniform distribution of Al and In in AlInN.

## Background

AlInN is a newly developed III-nitride for many promising applications due to its band gap being able to be tuned in a wide range of 0.70 ~ 6.14 eV with high spontaneous polarization [1-3]. AlInN/GaN heterojunction has been suggested as a strong candidate for high-power and high-frequency applications owing to the nearly lattice match (In = 0.17) and stress-free heterostructure in which the piezoelectric polarization charge is eliminated to reduce surface-related current collapse [4-8]. The attractive prospect for AlInN/GaN-based devices for high electron mobility transistors with advanced structure relies on high-quality AlInN epilayer. However, many properties of ternary AlInN alloys have not been well understood because of lack of high-quality films. Growth of AlInN is complicated by thermal control, which usually results in growth with composition inhomogeneities and phase separation [9-11]. Especially, the thermal instability may lead to phase separations and other defects which form on the substrate and may evolve during the growth resulting in rough and poorly uniform surface. The lattice-matched of high-quality AlInN can potentially lead to a reduction in threading dislocation and cracking as well as the elimination of strain-driven piezoelectric polarization field [12].

Various growth techniques have been used for growth of AlInN films, such as metal-organic chemical vapor deposition (MOCVD), radio-frequency molecular beam epitaxy, pulse laser deposition, and magnetron sputtering on *c*-plane sapphire substrates [13-15]. In the past few years, most of AlInN/GaN studies are done on films grown on sapphire substrate which has strong effects on the film properties, and among them, lattice-matched Al_1-*x*_In_*x*_N/GaN with 0.17 ~ *x* ~ 0.18 has received intensive attention [16-18]. The nearly lattice-matched AlInN layer grown on GaN/sapphire substrate usually contain some defects like hillocks, dislocations natively present in the GaN layer and the V-defects on GaN surface [19-21]. However, recent studied show that the growth of AlInN on GaN remains a challenge as many issues have to be addressed [22,23]. Hereinafter, we show that a high-quality and smooth Al_1-*x*_In_*x*_N film with *x* less than 0.17 can be epitaxially grown on GaN free-standing substrate with in-plane lattice match by employing MOCVD.

## Methods

In this work, we used hydride vapor phase epitaxy (HVPE)-grown GaN wafers as substrates which had a 1.5-in. diameter and 300-μm thickness with a full width at half maximum (FWHM) of the (0002) X-ray rocking curve (XRC) 100 arcsec (dislocation density approximately 1 × 10^7^ cm^-2^) and a root mean square (RMS) surface roughness of 1.23 nm. A 1.5-μm-thick homoepitaxial GaN film was firstly grown on GaN substrate at 970°C by MOCVD (Veeco Emcore D-180; Veeco Instruments Inc., Plainview, NY, USA) using TMGa and NH_3_ as the precursors for Ga and N to improve GaN crystallinity. After the growth of GaN layer, TMAl and NH_3_ were flowed into the chamber at 990°C for 1 min [24], followed by further growth of AlInN epilayer at temperature of 780 and 800°C. A TMAl/TMIn ratio of 1/10, a reactor pressure of 100 Torr, and a NH_3_ flow rate of 15,000 sccm were used for all AlInN growth.

The crystallinities of the samples were examined with high-resolution X-ray diffraction (XRD, Bruker D8; Bruker Corp., Billerica, MA, USA), and the surface morphologies were investigated by scanning electron microscopy (SEM, JEOL JSM-6500F; JEOL Ltd., Akishima, Tokyo, Japan) and atomic force microscopy (AFM, Veeco Innova). Structural characterization at atomic scale was performed in a JEOL JEM-ARM200F spherical aberration corrected scanning transmission electron microscope (STEM) in high-angle annular dark field (HAADF) imaging mode, operated at 200 kV. Cross-sectional transmission electron microscopy specimens were prepared in a focused ion beam system (FEI NOVA-200; FEI Company, Hillsboro, OR, USA) using a 30-kV Ga^+^ source.

## Results and discussion

**Figure 1:**
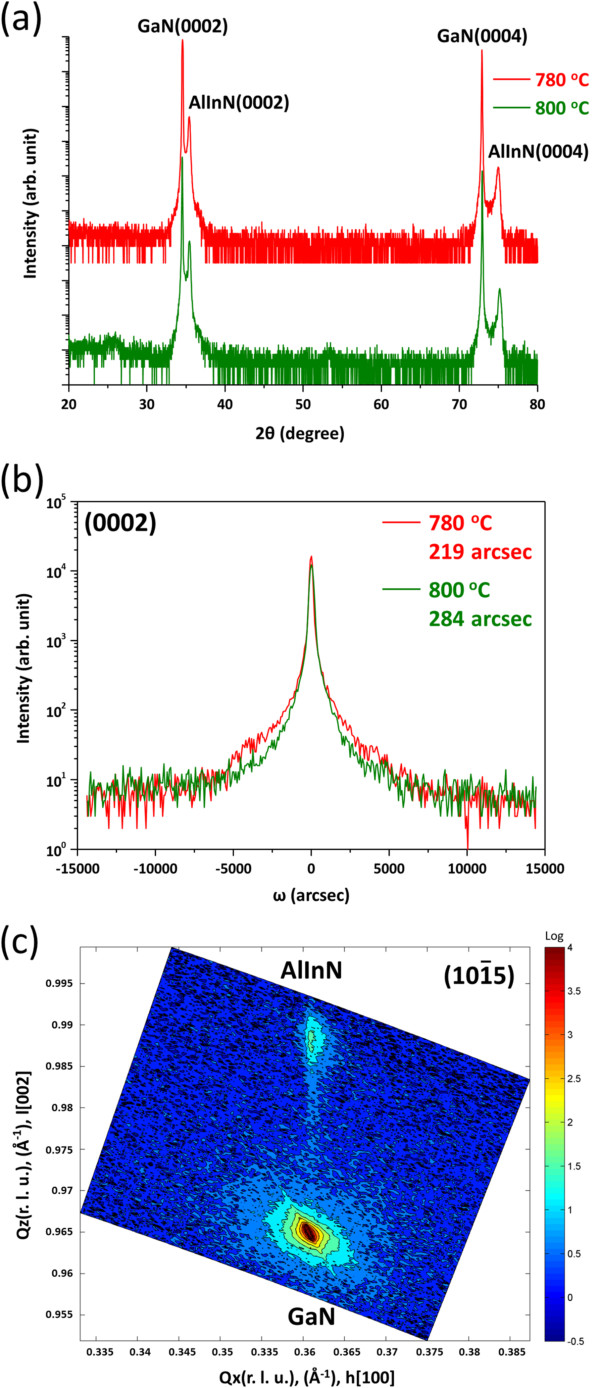
**XRCs and RSM showing crystallinities and coherency of the samples. (a)** θ/2θ scan XRD patterns of AlInN epilayer grown on GaN substrate at 780 and 800°C. **(b)** AlInN(0002) XRCs for the growth at 780 and 800°C showing FWHMs of 219 and 284 arcsec, respectively. **(c)**$\left(10\bar{1}5\right)$ RSM of the 780°C sample.

The crystallinities of the samples as examined with XRD can be seen in Figure 1a for the 780 and 800°C-grown AlInN film, in which Al_1-*x*_In_*x*_N exhibits only (0002)/(0004) reflections, suggesting that it is of single phase. By using Bragg’s law, the measured AlInN (0004) interplanar spacing can give *c* = 5.064 Å of the 780°C sample. From previous studies by Lorenz et al. [1] and Darakchieva et al. [25], the Al_1-*x*_In_*x*_N layer may have a composition with *x* approximately 0.13 which was determined by Rutherford backscattering spectroscopy and XRD. Also, the film has a better crystallinity with the (0002) XRC full width at half maximum (FWHM) of 219 arcsec as shown in Figure 1b than that of the 800°C-grown AlInN which can be shown *x* about 0.10. In previous studies of MOCVD growth, it has been shown that the high-temperature growth results in low In composition because of In desorption with temperature [26]. In Figure 1c, asymmetric reciprocal space mapping (RSM) shows that the reciprocal-lattice points of AlInN $\left(10\bar{1}5\right)$ and GaN $\left(10\bar{1}5\right)$ are well aligned in *Q*_*x*_ direction, i.e., in-plane lattice match, indicating pseudomorphic growth of AlInN on GaN [27].

**Figure 2:**
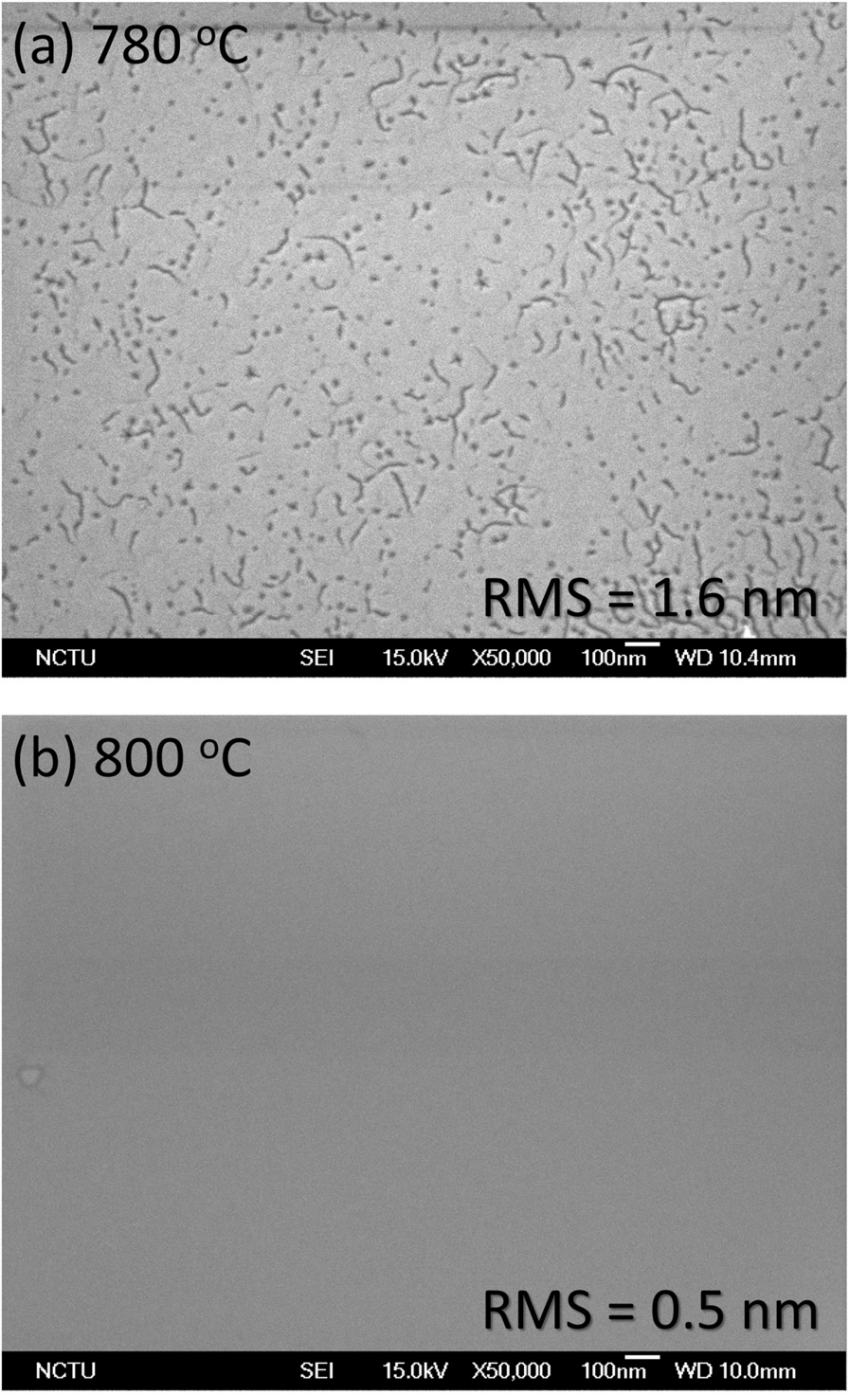
SEM images showing the surface morphologies of AlInN grown on GaN substrate at (a) 780°C and (b) 800°C.

The surface morphologies of the AlInN epilayers grown on GaN substrate by MOCVD were investigated with SEM and AFM. The surface of 800°C sample is much smoother than 780°C sample as shown in Figure 2. The RMS surface roughness for AlInN grown at 800°C as determined by AFM from 3 × 3 μm^2^ scanning area is 0.5 nm, compared with 1.6 nm for the 780°C sample. Similar observation has been reported by Ichikawa et al. that the higher growth temperature results in a smoother surface with a decrease of the In composition [28].

**Figure 3:**
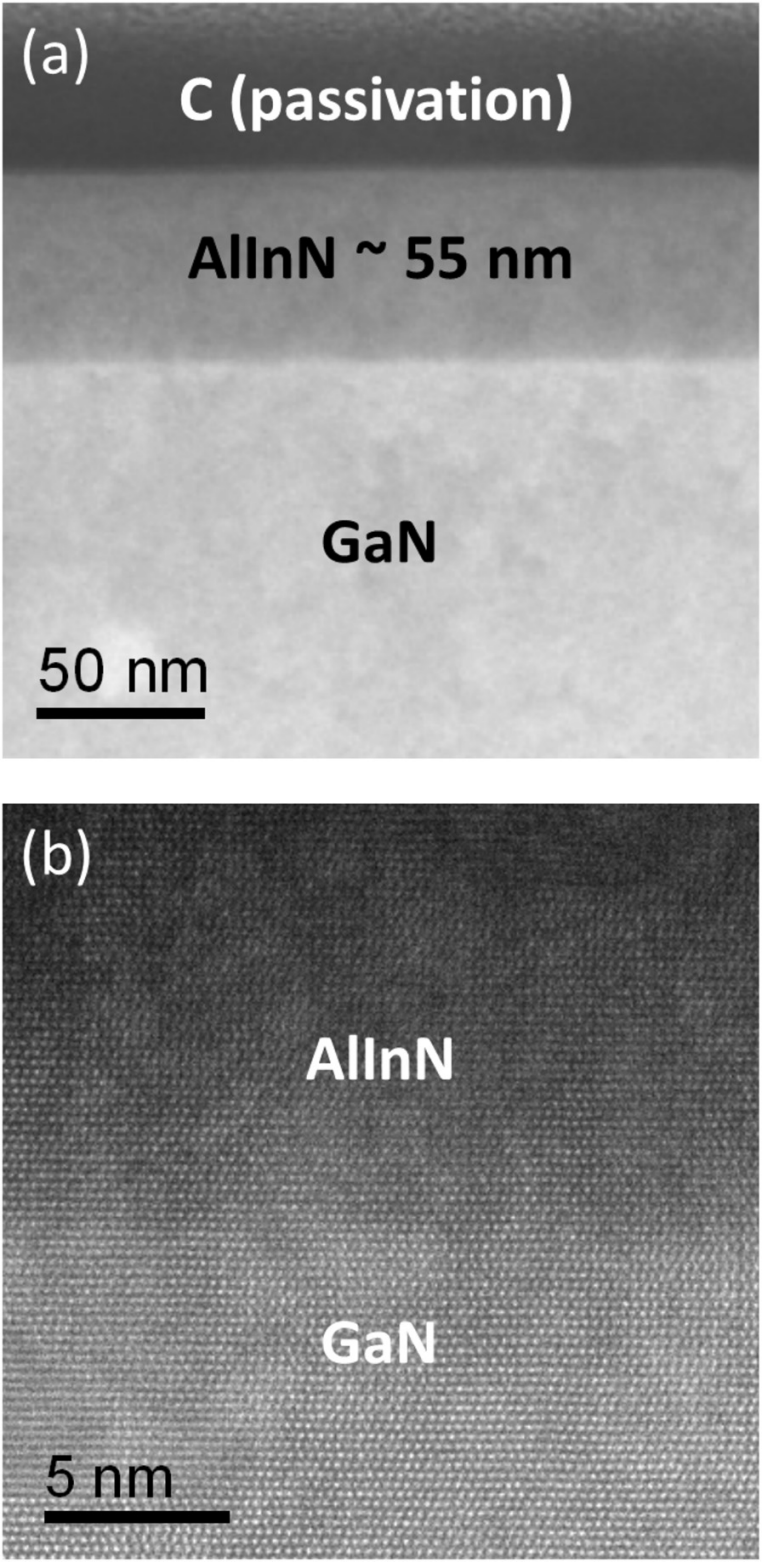
**STEM-HAADF Z-contrast images from the interface region of the 780°C sample in**$\left[11\bar{2}0\right]$**zone axis. (a)** Low-magnification cross-sectional image. **(b)** Atomic resolution image.

As the AlInN film is shown to be lattice-matched with GaN in RSM, the lattice images from high-resolution TEM are difficult to resolve the interface. Therefore, we used the STEM-HAADF images in *Z*-contrast for characterization of the interface and examination of the uniformity of the film. The HAADF images were acquired with the inner and outer collection semiangles of 68 and 174.5 mrad, respectively, and with a probe size of 1.5 Å. Figure 3a shows a low-magnification STEM-HAADF image of the 780°C sample in a cross-sectional view along the $\left[11\bar{2}0\right]$ zone-axis. As can be seen, the contrast of the GaN substrate is much brighter than that of the AlInN layer, consistent with the *Z*-contrast interpretation since the average atomic number is greater for GaN than Al-rich AlInN. Also, the image shows that the AlInN/GaN interface is sharp and the AlInN film is about 55 nm thick with a uniform distribution of Al and In in this ternary structure at nanometer scale. An atomic resolution STEM-HAADF image from the interface region is shown in Figure 3b. Each bright dot corresponds to the atomic column Al/InN and Ga in AlInN and GaN with invisible N atoms, and the intensity of Ga is much stronger than Al/In as expected. From the image contrast, both AlInN and GaN have reasonably uniform distributions around the interfacial region. Furthermore, it can be shown that the Al/In and Ga atomic arrangements are in excellent lattice match at the interface without any misfit dislocations, consistent with the above RSM result.

The above results show that lattice-matched Al_1-*x*_In_*x*_N films on GaN with *x* < 0.17 are of high crystallinity with quite smooth surface, which can be comparable with those of Al_1-*x*_In_*x*_N films grown on free-standing GaN substrates with *x* close to 0.17 [18].

## Conclusions

In this work, we successfully fabricated high-quality AlInN/GaN heterostructures by MOCVD. XRD, SEM, and STEM results reveal that the film is of good crystallinity and uniformity in composition, smooth surface flatness, and the abrupt heterointerface with lattice-matched AlInN epilayer on free-standing GaN substrate at atomic scale.

## Competing interests

The authors declare that they have no competing interests.

## Authors’ contributions

PYL carried out the XRD and STEM works and wrote the manuscript. JYC designed the thin-film deposition process and carried out the SEM work. YSS helped in the RSM measurements. LC conceived of the study and participated in its design and coordination with analysis and interpretation of data and the revision of the manuscript. All authors read and approved the final manuscript.

## References

[B1] Lorenz K Franco N Alves E Watson IM Martin RW O’Donnell KP Anomalous ion channeling in AlInN/GaN bilayers: determination of the strain state Phys Rev Lett 2006 97 085501 17026313 10.1103/PhysRevLett.97.085501

[B2] Rinke P Winkelnkemper M Qteish A Bimberg D Neugebauer J Scheffler M Consistent set of band parameters for the group-III nitrides AlN, GaN, and InN Phys Rev B 2008 77 075202

[B3] Gonschorek M Carlin JF Feltin E Py MA Grandjean N Darakchieva V Monemar B Lorenz M Ramm G Two-dimensional electron gas density in Al(1-x) In(x) N/AlN/GaN heterostructures (0.03 <= x <= 0.23) J Appl Phys 2008 103 093714 10.1063/1.2917290

[B4] Khan MA Bhattarai A Kuznia JN Olson DT High-electron-mobility transistor based on a GaN-AlxGa1-xN heterojunction Appl Phys Lett 1993 63 1214 1215 10.1063/1.109775

[B5] Someya T Werner R Forchel A Catalano M Cingolani R Arakawa Y Room temperature lasing at blue wavelengths in gallium nitride microcavities Science 1999 285 1905 1906 10.1126/science.285.5435.1905 10489367

[B6] Mishra UK Parikh P Wu YF AlGaN/GaN HEMTs - an overview of device operation and applications Proc IEEE 2002 90 1022 1031 10.1109/JPROC.2002.1021567

[B7] Trew RJ Bilbro GL Kuang W Liu Y Yin H Microwave AlGaN/GaN HFETs IEEE Microw Mag 2005 6 56 66

[B8] Sakalauskas E Behmenburg H Hums C Schley P Rossbach G Giesen C Heuken M Kalisch H Jansen RH Blasing J Dadgar A Krost A Goldhahn R Dielectric function and optical properties of Al-rich AlInN alloys pseudomorphically grown on GaN J Phys D Appl Phys 2010 43 365102 10.1088/0022-3727/43/36/365102

[B9] Hums C Bläsing J Dadgar A Diez A Hempel T Christen J Krost A Lorenz K Alves E Metal-organic vapor phase epitaxy and properties of AlInN in the whole compositional range Appl Phys Lett 2007 90 022105 10.1063/1.2424649

[B10] Gadanecz A Bläsing J Dadgar A Hums C Krost A Thermal stability of metal organic vapor phase epitaxy grown AlInN Appl Phys Lett 2007 90 221906 10.1063/1.2743744

[B11] Sadler TC Kappers MJ Oliver RA The effects of varying metal precursor fluxes on the growth of InAlN by metal organic vapour phase epitaxy J Cryst Growth 2011 314 13 20 10.1016/j.jcrysgro.2010.10.108

[B12] Dadgar A Schulze F Bläsing J Diez A Krost A Neuburger M Kohn E Daumiller I Kunze M High-sheet-charge-carrier-density AlInN/GaN field-effect transistors on Si(111) Appl Phys Lett 2004 85 5400 5402 10.1063/1.1828580

[B13] Guo QX Tanaka T Nishio M Ogawa H Structural and optical properties of AlInN films grown on sapphire substrates Jpn J Appl Phys 2008 47 612 615 10.1143/JJAP.47.612

[B14] Kim-Chauveau H de Mierry P Chauveau JM Duboz JY The influence of various MOCVD parameters on the growth of Al1-xInxN ternary alloy on GaN templates J Cryst Growth 2011 316 30 36 10.1016/j.jcrysgro.2010.12.040

[B15] Chen WC Wu YH Peng CY Hsiao CN Chang L Effect of In/Al ratios on structural and optical properties of InAlN films grown on Si(100) by RF-MOMBE Nanoscale Res Lett 2014 9 204 10.1186/1556-276X-9-204 24855462 PMC4012525

[B16] Butté R Carlin JF Feltin E Gonschorek M Nicolay S Christmann G Simeonov D Castiglia A Dorsaz J Buehlmann HJ Christopoulos S Baldassarri Höger von Hög G Grundy AJD Mosca M Pinquier C Py MA Demangeot F Frandon J Lagoudakis PG Baumberg JJ Grandjean N Current status of AlInN layers lattice-matched to GaN for photonics and electronics J Phys D Appl Phys 2007 40 6328 6344 10.1088/0022-3727/40/20/S16

[B17] Mouti A Rouviere JL Cantoni M Carlin JF Feltin E Grandjean N Stadelmann P Stress-modulated composition in the vicinity of dislocations in nearly lattice matched AlxIn1-x N/GaN heterostructures: a possible explanation of defect insensitivity Phys Rev B 2011 83 195309

[B18] Liu GY Zhang J Li XH Huang GS Paskova T Evans KR Zhao HP Tansu N Metalorganic vapor phase epitaxy and characterizations of nearly-lattice-matched AlInN alloys on GaN/sapphire templates and free-standing GaN substrates J Cryst Growth 2012 340 66 73 10.1016/j.jcrysgro.2011.12.037

[B19] Vennéguès P Diaby BS Kim-Chauveau H Bodiou L Schenk HPD Frayssinet E Martin RW Watson IM Nature and origin of V-defects present in metalorganic vapor phase epitaxy-grown (InxAl1-x) N layers as a function of InN content, layer thickness and growth parameters J Cryst Growth 2012 353 108 114 10.1016/j.jcrysgro.2012.05.004

[B20] Perillat-Merceroz G Cosendey G Carlin JF Butté R Grandjean N Intrinsic degradation mechanism of nearly lattice-matched InAlN layers grown on GaN substrates J Appl Phys 2013 113 063506 10.1063/1.4790424

[B21] Potin V Gil B Charar S Ruterana P Nouet G HREM study of basal stacking faults in GaN layers grown over sapphire substrate Mat Sci Eng B Solid 2001 82 114 116 10.1016/S0921-5107(00)00709-1

[B22] Schenk HPD Nemoz M Korytov M Vennéguès P Dräger AD Hangleiter A Indium incorporation dynamics into AIInN ternary alloys for laser structures lattice matched to GaN Appl Phys Lett 2008 93 081116 10.1063/1.2971027

[B23] Buβ ER Rossow U Bremers H Hangleiter A Lattice-matched AlInN in the initial stage of growth Appl Phys Lett 2014 104 162104 10.1063/1.4872226

[B24] Lin PY Chen JY Chen YC Chang L Effect of growth temperature on formation of amorphous nitride interlayer between AlN and Si(111) Jpn J Appl Phys 2013 52 08JB20 10.7567/JJAP.52.08JB20

[B25] Darakchieva V Beckers M Xie MY Hultman L Monemar B Carlin JF Feltin E Gonschorek M Grandjean N Effects of strain and composition on the lattice parameters and applicability of Vegard’s rule in Al-rich Al(1-x) In(x) N films grown on sapphire J Appl Phys 2008 103 103513 10.1063/1.2924426

[B26] Yakovlev EV Lobanova AV Talalaev RA Watson IM Lorenz K Alves E Mechanisms of AlInN growth by MOVPE: modeling and experimental study Phys Status Solidi C 2008 5 1688 1690 10.1002/pssc.200778588

[B27] Lorenz K Franco N Alves E Pereira S Watson IM Martin RW O’Donnell KP Relaxation of compressively strained AlInN on GaN J Cryst Growth 2008 310 4058 4064 10.1016/j.jcrysgro.2008.07.006

[B28] Ichikawa J Sakai Y Chen ZT Fujita K Egawa T Effect of growth temperature on structural quality of InAlN layer lattice matched to GaN grown by metal organic chemical vapor deposition Jpn J Appl Phys 2012 51 01AF07 10.7567/JJAP.51.01AF07

